# Hemoglobin induced cell trauma indirectly influences endothelial TLR9 activity resulting in pulmonary vascular smooth muscle cell activation

**DOI:** 10.1371/journal.pone.0171219

**Published:** 2017-02-02

**Authors:** Zoe Loomis, Paul Eigenberger, Katherine Redinius, Christina Lisk, Vijaya Karoor, Eva Nozik-Grayck, Scott K. Ferguson, Kathryn Hassell, Rachelle Nuss, Kurt Stenmark, Paul Buehler, David C. Irwin

**Affiliations:** 1 Cardiovascular and Pulmonary Research Laboratory, Department of Medicine, University of Colorado Denver, Anschutz Medical Campus, Aurora, Colorado, United States of America; 2 Division of Hematology and Colorado Sickle Cell Treatment and Research Center, University of Colorado-Denver School of Medicine, Aurora, Colorado, United States of America; 3 Division of Hematology, The Center for Biologics Evaluation and Research, United States Food and Drug Administration, Bethesda, Maryland, United States of America; Augusta University, UNITED STATES

## Abstract

It is now well established that both inherited and acquired forms of hemolytic disease can promote pulmonary vascular disease consequent of free hemoglobin (Hb) induced NO scavenging, elevations in reactive oxygen species and lipid peroxidation. It has recently been reported that oxidative stress can activate NFkB through a toll-like receptor 9 (TLR9) mediated pathway; further, TLR9 can be activated by either nuclear or mitochondrial DNA liberated by stress induced cellular trauma. We hypothesis that Hb induced lipid peroxidation and subsequent endothelial cell trauma is linked to TLR9 activation, resulting in IL-6 mediated pulmonary smooth muscle cell proliferation. We examined the effects of Hb on rat pulmonary artery endothelial and smooth muscle cells (rPAEC and rPASMC, respectively), and then utilized TLR9 and IL6 inhibitors, as well as the Hb and heme binding proteins (haptoglobin (Hp) and hemopexin (Hpx), respectively) to further elucidate the aforementioned mediators. Further, we explored the effects of Hb *in vivo* utilizing endothelial cell (EC) specific myeloid differentiation primary response gene-88 (MyD88) and TLR9 null mice. Our data show that oxidized Hb induces lipid peroxidation, cellular toxicity (5.5 ± 1.7 fold; p≤0.04), increased TLR9 activation (60%; p = 0.01), and up regulated IL6 expression (1.75±0.3 fold; p = 0.04) in rPAEC. Rat PASMC exhibited a more proliferative state (13 ± 1%; p = 0.01) when co-cultured with Hb activated rPAEC. These effects were attenuated with the sequestration of Hb or heme by Hp and Hpx as well as with TLR9 an IL-6 inhibition. Moreover, in both EC-MyD88 and TLR9 null mice Hb-infusion resulted in less lung IL-6 expression compared to WT cohorts. These results demonstrate that Hb-induced lipid peroxidation can initiate a modest TLR9 mediated inflammatory response, subsequently generating an activated SMC phenotype.

## Introduction

Hemolysis induced release of hemoglobin (Hb) occurs in a host of patient populations including those suffering from hemolytic anemia[1], severe sepsis[2], or those prescribed chronic renal replacement therapy (CRRT) or extracorporeal membrane oxygenation (ECMO)[3]. Unless captured, sequestered, and compartmentalized by scavenging-proteins, Hb’s catalytic iron and globin chains become pathological mediators that contribute to morbidity associated with progression of lung and vascular diseases [4]. For example, if not neutralized, Hb contributes to acute lung injury (ALI) from sepsis [2, 5], as well as, the more chronic vascular disease of pulmonary hypertension (PH) in sickle cell disease (SCD) [6–8]

Pulmonary vascular inflammation is a key mediating factor in the development and progression of hemolytic anemia associated PH; however, not all of the mechanisms by which extracellular Hb promotes vascular inflammation and its contribution to PH have been clearly elucidated. One putative explanation suggests that cell free Hb can impact the progression of PH by its rapid reaction with NO [1, 6–9]. More specifically, once released from the red blood cells, Hb extravasates from the vessel lumen into the tissue compartment and reacts quickly with NO and/or other reactive oxygen intermediates within the microenvironment. This results in the reduction of NO bioavailability and an induction of pulmonary artery vasoconstriction [7, 8, 10]. Moreover, it is also recognized that there are other Hb mediated mechanisms which potentiate the vascular remodeling associated with the deadly progression of PH [11].

It is now well established that Hb-mediated reactions with peroxides that lead to heme release, tissue iron accumulation, cellular lipid peroxidation, necrosis and/or tissue damage, can also play a commanding role in the pathophysiological outcomes in the pulmonary microenvironment. The resultant tissue damage from these events may be more pronounced when Hb is driven to higher oxidation states (ferric- HbFe^3+^ and ferryl- HbFe^4+^), as occurs in local environments that have conditions of increased cellular hydrogen peroxide (H_2_O_2_) formation. Such environments include areas of pre-existing inflammation or tissue hypoxia, in which, the biochemical reaction between deoxy-HbFe^2+^ and H_2_O_2_ creates oxo-ferryl^4^, Hb [Hb(Fe^4+^ = O)], ferric Hb [Hb(Fe^3+^)], and the formation of the protein radical [•Hb(Fe^4+^ = O)] [4]. These reactions perpetuate reactive oxygen species formation and accelerate Hb protein unfolding, intermolecular crosslinking and progressive degradation of the Hb molecule into precipitated protein, heme and iron. For additional detail on these biochemical reactions and their consequences we refer the reader to the review by Smith and McCulloh [12]. Importantly, excessive Hb and peroxides can promote a local environment rich in Danger Associated Molecular Patterns (DAMPs) that activate Toll-Like Receptors (TLRs). These events are capable of eliciting a vascular inflammatory response that may initiate or exacerbate the progression and symptoms of PH [13, 14].

The TLR family is a class of 11 proteins that are expressed on a variety of cell types, including the vascular endothelium, and play a key role in the innate immune response by recognizing pathogen- and danger-associated molecular patterns (PAMPs / DAMPS)[15]. In contrast to PAMPs released from infectious pathogens, DAMPS are endogenous molecules that can initiate or perpetuate an inflammatory response[16]. Recently it has been recognized that mitochondrial or nuclear CpG DNA released due to tissue trauma can stimulate TLR9 as DAMPs [17–19]. It’s also been suggested that the TLR9 receptor may be an earlier sentinel for the vascular immune interface by detecting damaged mitochondrial DNA that have been excised and translocated to the cytosol[20]. However, to our knowledge there have been no investigations testing the role of a TLR-9 pathway in the potentiation of pulmonary hypertension secondary to Hb mediated inflammation. Thus, we hypothesized that vascular cell damage incurred from Hb mediated redox reactions may be a unique source for rogue mitochondrial or nuclear CpG DNA motifs that subsequently trigger a TLR9-regulated inflammatory response contributing to hemolytic anemia associated PH. Our data supports this provocative idea that Hb-mediated endothelial death or damage contributes to pulmonary vascular disease through a TLR9 mechanism.

## Materials and methods

### Animals

Male and female C57Bl/6 wildtypeMyD88 floxed mice (B6.129 (SJL)-MyD88^tm1Defr^/J), and Tek-Cre (B6.Cg-Tg (Tek-cre) 12Flv/J) mice were obtained from a commercial vendor (Jackson Laboratories, Bar Harbor, ME, USA). MyD88f/f mice were bred with TekCre (+) mice to generate endothelial cell specific MyD88 knockout mice. TLR9 KO mice were gifted to us from Bonn University (Germany). Transnetyx performed the genotyping of mice used for breeding and experiments as per Jackson Labs protocols. All experimental protocols were reviewed and approved by the Institutional Animal Care and Use Committee at the University of Colorado Denver Anschutz Medical Campus. All study animals survived the surgical procedure and indwelling catheterization into the jugular vein. None of the mice exhibited any signs or symptoms indicative of systemic infection. After surgery, wounds healed within 7 days.

### Hemoglobin

Purified human endotoxin-free Hb (lipopolysaccharide <0.5 endotoxin units) was prepared from outdated blood as previously described[21]. Catalase and superoxide dismutase were removed using an AKTA FPLC system and an XK 16/70 column containing Superdex 200 medium or an XK 50/100 column containing Sephacryl 200 medium (GE Healthcare Bio-Sciences, Piscataway, NJ, USE) as previously described[13]. Several runs were pooled and concentrated to 200 mg/mL using Centricon Plus– 70 with 30 kDa cutoff membrane filters (Millipore, Bilerica MA, USA). The starting composition of Hb was 96.5 ± 1.3% Fe^2+^, 3.50 ± 0.23% Fe^3+^ and no measurable hemichrome. Endotoxin concentration was <0.5 EU/mL as determined by the limulus ambocyte lysate (LAL) assay, and failed to activate macrophages in culture.

### Indwelling catheter placement and tether system

Adult mice (20–25 g) were provided bupenorphrine (0.1 mg/kg; 100 ul; SQ, bid) 12 h prior to and for 48 h post catheter placement. Animals were anesthetized with isoflourane starting at 4% then brought down to 2–3% for the surgery. The neck and the area dorsally between the scapulae were shaved and the areas scrubbed with a Betadine solution. Indwelling catheters were placed in the jugular vein and tunneled subcutaneously and exteriorized between the shoulder blades. While still anesthetized, the animals were placed in a rubber harness and connected by a flexible tether to a swivel suspended above the cage (SAI, USA). The swivel has a counter-balance mounted on top of the cage allowing unrestrictive movement. Post surgery, all animals received a bolus of 100 ul of saline via the jugular catheter prior to connecting to the pump. Bupivicaine (20 ul) was injected into the wound before it was stapled closed and a topical antibiotic was applied to the skin. The animal was maintained on a heating pad and monitored until recovered from anesthesia. Post operative support also included closely monitoring the animal for 36 h to ensure eating, drinking, and grooming have returned to normal. If an animal lost more than 15% of his presurgical body weight, it was sacrificed (150 mg/kg sodium pentobarbitol I.P.).

As expected 12% of the animals (n = 5) died prior to completing the study protocol, and were not included in the data analysis. These deaths were attributed to, post operative complications due to blood loss during surgery, and poor recovery from analgesia.

The animals were recovered for 7 days after surgery. Catheter patency was maintained with an infusion of 9uL/hr of heparinized saline during the recovery period. Wild Type, EC-MyD88^-/-^, and TLR9^-/-^ mice (n = 12 per strain) were randomized into two groups, receiving either 0.9% saline (n = 6 per strain) or 6mg Hb per day (~28 υg •υl^-1^•hr^-1^; n = 6 per strain) for 21 days. During the infusion, all animals had unrestricted access to food and water. At the end of study protocol animasl were humanly euthanized with intraperitoneal injection of pentobarbital (150 mg/kg)

### Materials

The following antibodies were used: IL6 (Cat # ab9342), 1:500 (Abcam); βactin (Cat # ab8226), 1:1000 (Abcam); TLR9 (Cat # GTX59899), 1:1000 (Genetex). Superoxide and catalase free human HbA was prepared as previously described [21]. Haptoglobin was provided by Bio Products Laboratory Ltd. and contained primarily Hp 1–1 (dimeric form) (Elstree, United Kingdom), and Hemopexin was purchased from Sigma Aldrich (St. Louis, MO). Hb-Hp complexes were prepared as previously described[21]. Briefly, to prepare Hb–Hp complexes for *in vitro* assays, HbFe2^+^ was added to Hp in a 2:1 ratio (1.2ml (120mg) Hb to 1ml (60mg)Hp). Free Hb was removed from the Hb–Hp complex by injecting 5ml of mixture onto a semi preparative BioSep-SEC-S3000 (600 mm x 21.2mm) column (Phenomenex,Torrance,CA,USA) with 50mM potassium phosphate, pH7.4, as the mobile phase and monitored at an absorbance λ 280 and 405nm. Samples of Hb–Hp eluted at a 12- to 17-min elution time and were collected, pooled, and concentrated using centricon plus 70 (100 KDa cutoff) centrifugal filters to a heme concentration of 50 mM. All Hb concentrations for free and bound Hb are expressed as Hb (μM heme). Rat pulmonary artery endothelial cells (rPAEC) were cultured under normal cell culture conditions, in DMEM media plus 10% FBS were untreated (control) or treated with HbFe^2+^ (1μM to 25 μM heme) between 4 and 24 hours (as noted in figure legends) in the presence or absence of: **(1)** Glucose oxidase (Sigma; Cat # G7141-50KU); **(2)** Super oxide dismutase and catalase (150 units ea.; Sigma; Cat # S5395, C6665, respectively); **(3)** TLR9 inhibitor 4084 (Invivogen, Cat # tlrl-4084); **(4)** DNAase (New England Biolabs, Cat # M0303L); **(5)** IL6 neutralizing antibody (Abcam; Cat # ab9324); **(6)** Hb bound Haptoglobin (Hb-Hp); or **(7)** Hemopexin (25 υM). When appropriate, cells were also treated with CpG DNA (25 Inivogen; CpG SL01; Cat # tlrl-ds101) as positive control for TLR9 activation. All treatments were added to the cell culture medium at the time of Hb treatments. After 6–24 h of Hb exposure, TLR9 activity, lipid peroxidation, supernatant lactate dehydrogenase, nucleic acid concentrations, cleaved TLR9 protein, IL-6 mRNA, and pulmonary smooth muscle cell activation were determined. All experiments were repeated at least three times on at least two separate days (n = 6).

### Cell lines

HEK-Blue TLR9 reporter cells (Invivogen; Cat # HEK-Blue hTLR9, HEK-Blue Null I) are designed to study TLR9 stimulation by the activation of NFkB and AP-1. Where HEK-Blue Null I are TLR9 deficient cells designed to determine any unspecific TLR9 activity. HEK cells were cultured under standard cell culture conditions per manufactures instructions. Briefly hTLR9 cell media was comprised of DMEM media, 10% FBS serum, penicillin (50 U/ml), streptomycin (50 υg/ml) normocin (100 υg/ml), zeocin (100 υg/ml), and blasticidin (10 υg/ml). HEK null cells utilized the same media with the exception of blasticidin. All HEK reporter cell experiments were completed in 24-well polystyrene plates (Fisher; Cat # 08-772-1) seeded at a concentration of 40,000 cells per well. HEK blue colorimetric assays were evaluated for color absorption at 655 nm on a synergy 2 bioteck plate reader. To avoid Hb interference with the colorimetric assay after 24 h of exposure to Hb or vehicle control all cells were washed twice with PBS and HEK blue media was added for 2 h prior to assessing color absorption. To rule out any unspecific TLR9 HEK-Blue activation, all HEK-blue assays were run in duplicated wells seeded with hTLR9 and corresponding HEK-blue null cells. Values from hTLR9 samples were normalized to the corresponding well of HEK-blue Null cells or hTLR9/HEK blue null). Primary rat pulmonary artery endothelial (rPAEC) cells were provided by T Stevens (University of Southern Alabama, USA), and cultured as previously described [22].

### Lipid peroxidation analysis using Click-iT chemistry

Rat PAECs were seeded at 10,000 cells/cm2 in four-well, 1.0mm, glass chamber slides (Electron Microscopy Sciences, Hatfield, PA, USA) and grown to confluence. Cells were treated with DMEM (Sigma-Aldrich) containing 0.2% FBS, 50mM Click-iT linoleamide alkyne (Life Technologies) and exposed to 6.25 υM superoxide dismutase/catalase-depleted human HbFe^2+^ (25 υM heme) in the presence or absence of human Haptoglobin and Hemopexin. Cells were exposed to these treatments for 6 and 16h time points and then immediately washed with PBS and fixed in 3.7% formalin for 10 min at room temperature (all subsequent steps were performed at room temperature). Cells were permeabilized with 0.5% Triton X-100 for 10 min and blocked with 1% bovine serum albumin for 30 min. After three washes in PBS, the cells were stained with the Click-iT reaction cocktail for 30 min in the dark. After three washes in PBS, a nuclear stain was performed using Hoechst 33342 (2mg/mL). Imaging was conducted on a Nikon Eclipse T*i*-E inverted epifluorescence microscope (Nikon Instruments). The percentage fluorescence was quantified using the Fiji distribution of ImageJ. Briefly, a threshold analysis was used to select stain-positive pixels on 60x magnification images. Nuclei were excluded by adjusting the hue to exclude blue wavelengths. The stain-positive pixel area was measured and compared to total area of the image. The process was repeated using up to seven separate images per group.

### Cytotoxicity detection (LDH)

For all experiments, rPAEC were used from the same passage and grown during the same time frame to confluence in culture media containing 10% FBS, unless otherwise specified. Cells were plated on a 96 well, clear, flat bottom cell culture plate at a concentration of 1250 cells/well. Treatments were run in triplicate including the following controls: background control (media without cells), low control, and high control (as per manufacturer’s instructions). Cells were treated with DMEM (Sigma-Aldrich) containing 0.2% FBS and exposed to 6.25υM superoxide dismutase/catalase-depleted human HbFe^2+^ in the presence or absence of human Haptoglobin and Hemopexin. The treatments were incubated on the cells for 6 or 16 hrs. The manufacturer’s instructions were used for the subsequent steps and absorbance changes were measured at 490 or 492 nm by a microplate reader (BioTek Instruments, Winooski, VT). To determine the percent cytotoxicity, the following calculations were performed:

Cytotoxicity (%) = [(exp value – low control)/(high control – low control)] × 100

### Western blot analysis

Protein was isolated from cell suspensions of rPAEC or whole lung lysates. Briefly, NP40 lysis buffer with proteinase K inhibitors were placed on the cells or tissue. Tissue was disrupted by homogenization and cells were scraped from the plate at 4°C, and concentrations were determined using a Pierce BCA Protein Assay. Briefly, analysis was performed using 15 mg of sample protein run under denaturing and reducing conditions on Biorad Criterion Tris-HCl 12.5% gels with a Biorad SDS-Page blot system. TLR9 cleaved and full length bands were normalized to βactin. Statistical analysis was determined from the fold difference from NT (no treatment) control on the same gel. Gels were imaged on an Alpha Innotech gel documentation system (Protein Simple, Santa Clara, CA, USA) and densitometric analyses were performed using ImageJ software (version 1.44o, National Institutes of Health, USA)

### qtPCR for IL-6

RNA was isolated from cell suspensions of rPAEC using Qiagen RNeasy Plus Mini Kit with gDNA filter column (Qiagen, Valecia, CA, USA). RNA was quantified spectrophotometrically (NanoDrop 1000), and equal amounts (1 ug) were loaded with Quanta cDNA Supermix (Quanta Biosciences, Gaithersburg, MD, USA) for cDNA synthesis. cDNA was diluted 1:10 in TE buffer for downstream reactions. Primers for rat-IL6 (Qiagen QuantiTect QT00182896) and rat-ACTB (Qiagen QuantiTect QT00193473) were used in reactions with 100ug cDNA and Quanta PerfectCT a SYBR Green Master Mix. Ninety-six-well PCR plates were run Applied Biosystems AB7300 (ThermoFisher, Waltham, MA, USA), with initial denaturing (95°C, 2 min) followed by 40 cycles of denaturing (95°C, 15s), annealing (55°C, 30s), and extension (72°C, 30s). A melting curve was run to confirm specificity of PCR products. Experimental and reference genes were run on the same plates and relative quantification determined by the ΔC_T_ method.

### Endothelial and smooth muscle cell co-culture system

For all experiments, rPAEC and rPASMCs were used from the same passages (5–10) and grown during the same time frame to confluence in standard cell culture conditions using culture media containing 10% FBS, unless otherwise specified. rPASMC were seeded at a concentration of 20,000 cells/well in the bottom well and rPAEC were seeded at a concentration of 10,000 cells/well onto 6.5 mm diameter polyethylene terephthalate inserts with a pore size of 3mM(BD Falcon, 351183). Cells were grown for 24 hours, quiesced for an additional 24 hours, and then exposed to HbFe^2+^, TLR9 inhibitor 4084, CpG SL01, or an IL6 neutralizing antibody for 24–30 h. We chose this time frame on the basis of when we observed TLR9 activity and increased IL-6 mRNA. Smooth muscle cell activation was evaluated using the Promega CellTiter 96^®^ Aqueous One Solution Cell Proliferation Assay (catalog number G5421). Differences in the absorbance spectrum were measured at 490nm by a microplate reader (BioTek Instruments, Winooski, VT) at 0 and 180 min time points. Quantitative analysis was performed by identifying the Δ of the two time points for each treatment group, the values of which were then compared back to the no treatment control. Data is reported as percent change from control.

### Right ventricular systolic pressure (RVSP) measurements

Mice were anesthetized with inhaled isoflurane (2%-5%). Right ventricular (RV) pressure were measured in a closed chest via a direct RV puncture. A 25-gauge needle attached to a pressure transducer, was introduced into the RV and placement confirmed by a live pressure tracing. Pressures were captured with the Cardiomax III Cardiac Output Computer (Columbus Instruments) connected to a Dell laptop computer running the Cardiomax III program v2.10. Systolic RV pressures (RVSP) were monitored for 30 seconds, and averaged every 10 seconds to account for beat to beat variability.

### Mouse IL 6 western blot assay

Interleukin 6 was measured in whole lung lysates using a western blot technique as describe above in “western blot analysis”. Briefly, Tissue (mouse lung) was lysed in a 1:100 dilution of NP40 buffer and Halt Protease Inhibitor cocktail (Thermo scientific #1862209). Homogenized samples were centrifuged at 13 K rpm for 10 minutes at room temperature; supernatant was used to assay protein levels. Primary antibodies against IL-6 and B-actin were purchased from Abcam. A 1:500 dilution of Rabbit anti-IL6 antibody (ab6672) in 5% NFDM and TBST buffer was incubated at 4°C for 72 hours. A 1:1000 dilution of Mouse anti-B-actin antibody (ab8226) in 5% NFDM and TBST buffer was incubated at 4°C overnight. Secondary antibodies purchased from Vector, peroxidase labeled anti-mouse IgG (H+L) (PI-2000) and peroxidase labeled anti-rabbit IgG (H+L) (PI-1000), a 1:1000 dilution in 5% NFDM and TBST buffer incubated for 1 hour at room temperature. The blot was visualized by chemiluminescent substrate SuperSignal west Femto (Thermo scientific #34096) exposed for 1 minute. Densitometry was performed using Image J (version 1.44o, National Institutes of Health, USA). Software.

### Morphology and immunohistochemistry of lung tissue

Five-micron sections of formalin–fixed, paraffin-embedded lung tissue were stained with hematoxylin and eosin by standard procedures at the University of Colorado Histology Core to assess vessel wall thickness. Immunohistochemistry analysis for α-smooth muscle actin, 4-hydroxynonenal, interlukin-6 (IL-6) was performed per the manufacturer’s instructions. Antigen retrieval was performed on serial lung sections and then incubated with antibodies against smooth muscle actin (1:500 abcam, ab5694, Cambridge, MA), 4HNE (1:500 abcam, ab46545 Cambridge, MA) and IL-6 (1:50, Santa Cruz, sc-1265). Lung sections were incubated with a fluorescent secondary antibody (either Alexa Fluor 488 goat anti-rabbit IgG (1:300 or 1:800; A11008) or Alexa Fluor 555 (1:500; A21422) Invitrogen, Carlsbad, CA). Primary antibodies were incubated at 4°C overnight, and Secondary antibody was incubated for 1 hour at room temperature. Slides were then mounted with Vectashield mounting medium for fluorescence with dapi (H-1200) from Vector and coversliped. Pulmonary vessels (outside diameter ~100 μm) were assessed for the presence of smooth muscle actin, 4HNE and IL-6 protein expression on a Nikon Eclipse T*i*-E inverted epi-fluorescent microscope (Nikon Instruments, Tokyo Japan).

### Statistical analysis

All experiments followed a randomized block design with the use of cells from at least three different cell preparations. In experiments with only two test groups significance was determined by students t-test. For multiple groups compared at a single time point significance was determined by one-way analysis of variance, and for experiments that time was considered a factor a two-way (group*time) analysis of variance was completed. Post-hoc analyses were completed with the Tukey-Kramer multiple comparison tests. Statistical analysis was completed using either the statistical software package JMP (version 5 SAS Institute, Cary, NC), or GraphPad (version 6.0) Statistical significance was defined as p ≤ 0.05.

## Results

### Hb activates TLR9 in HEK reporter cells in time and dose dependency

**Fig 1:**
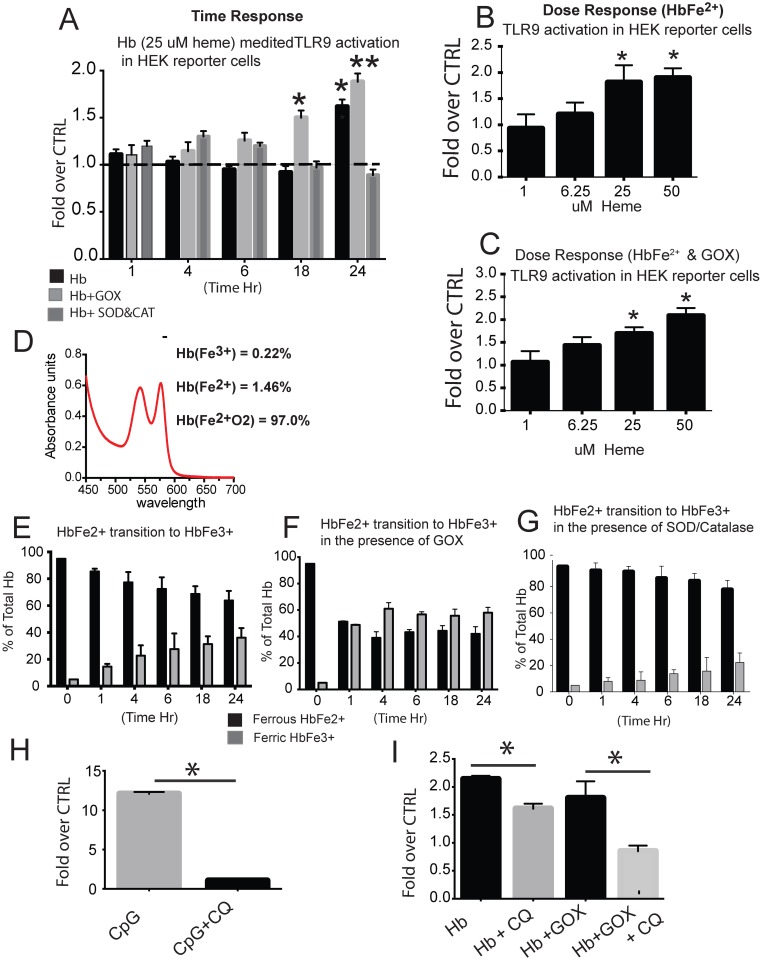
HEK-blue TLR9 reporter cell system. (A) Time response of TLR9 activity in HEK cells exposed to HbFe^2+^ (25 uM heme) HbFe^2+^ + GOX, or HbFe^2+^ + SOD & CAT (150 units). (B) Dose response to TLR9 activity in HEK cells exposed for 24 h to HbFe^2+^ (1–50 uM heme). (C) Dose response to TLR9 activity in HEK cells exposed to HbFe^2+^ + GOX. (D) Spectral analysis of catalase free HbFe^2+^ at time zero. (E,F,G) The percent of ferrous (HbFe^2+^) and ferric (HbFe^3+^) in the culture supernatant between 1 and 24 h for HEK reporter cells exposed to HbFe^2+^, HbFe^2+^ + GOX, or Hb Fe^2+^ + SOD & CAT. (H) HEK TLR9 reporter cells treated for 24 h with CpG (20 ug/ml) in the presence or absence of chloroquine. (I) HEK TLR9 reporter cells treated with ferrous Hb (25 uM heme) with and without GOX in the presence or absence of chloroquine. * p<0.01 vs. CTRL or chloroquine treated cells. **p = 0.008 vs CTRL. Hb- hemoglobin, GOX- glucose oxidase, SOD- superoxide dismutase, CAT- catalase. Experiments were completed with a minimum of 8 individual cell preparations per group / per day and run on two separate days (n = 16 per group).

To begin to understand the process by which HbFe^2+^ activates TLR9, we utilized a HEK blue reporter cell system and incubated HbFe^2+^ either alone or in the presence of an oxidant or antioxidant and sought to determine TLR9 activity between 1 and 24 h. Compared to vehicle control, we observed increased TLR9 activity at 24 h of Hb exposure, and at 18 and 24 h exposure of Hb plus glucose oxidase (Fig 1A). In contrast, we did not observe any increase in TLR9 activity when Hb was co-incubated with catalase and superoxide dismutase (Fig 1A). A dose response at 24h demonstrated the minimum effective dose of Hb to induce TLR9 activity in the presence of GOX is (25uM) (Fig 1B and 1C).

Inspection of the Hb oxidation states in HEK cell culture supernatant by spectrophotometry analysis revealed a steady increase of oxidized HbFe3+ reaching ~40% of the total by 24 h (Fig 1E). As expected, when Hb was incubated with glucose oxidase there was rapid oxidation (~50%) to HbFe3+ within the first hour, and was maintained at 60% between 4 and 24 h (Fig 1F). As predicted, incubation of Hb with catalase and superoxide dismutase attenuated the steady rise of oxidized Hb allowing only 20% to be converted to Fe3+ state at 24 h (Fig 1G).

To confirm the accuracy of our TLR9 reporter system, we exposed hTLR9 or HEK-blue TLR9 null cells for 24 h to CpG DNA (TLR9 agonist), HbFe^2+^ or HbFe^2+^ plus GOX in the presence or absence of chloroquine, a known TLR9 inhibitor [23]. As expected, we observed a robust TLR9 response in cells exposed to CpG DNA (Fig 1H), which was attenuated with co-treatment of chloroquine (Fig 1H and 1I) demonstrating our reporter cell system was accurately detecting TLR9 activity.

### Hb increases cleaved TLR9 expression in rat pulmonary artery endothelial cells

**Fig 2:**
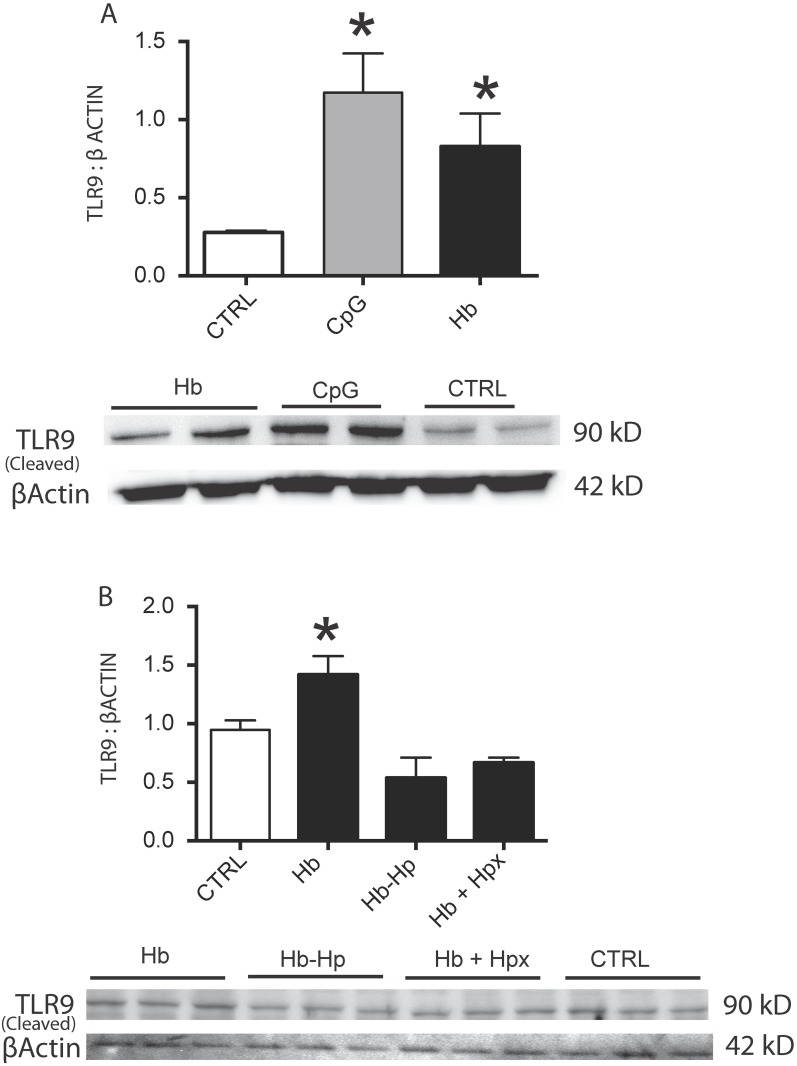
Rat PAEC cleaved TLR9 expression. (B) western blot for the cleaved TLR9 product in rat PAEC cells exposed for 24 h with either CpG DNA (20 ug/ml) or Hb (25 uM heme). (B) western blot for the cleaved TLR9 product in rat PAEC exposed to Hb, Hb bound to Hp (Hb-Hp), or co-treated with Hb plus hpx (Hb+Hpx). * p<0.04 vs ctrl. Hb- hemoglobin Hp-haptoglobin, Hpx- hemopexin. Experiments were completed with a minimum of 2 individual cell preparations per group on four separate days (n = 8).

Next we sought to determine if TLR9 activation occurred in a more relevant vascular cell type other than our HEK reporter cell system, and if the Hb and heme binding proteins haptoglobin (Hp) or hemopexin (Hpx) at equimolar ratios to Hb attenuated this response. To this end, rPAEC were incubated for 24 h with either CpG DNA (positive control), Hb (25 uM heme), Hb bound to Hp (Hb-Hp; 25 uM heme), or co-treated with Hb and Hpx (Hb+Hpx) and analyzed for the functionally active cleaved portion of TLR9 protein[24]. At 24 h, we observed CpG DNA and Hb induced increases of protein expression of the cleaved TLR9 (Fig 2A). Further, cleaved TLR9 expression was attenuated in cells incubated with either Hb-Hp or co-treated with Hb+Hpx (Fig 2B).

Taken together these initial studies in our HEK reporter cell system and rPAEC suggest that either the oxidation process or by-products from oxidized Hb, such as heme, are capable of inducing TLR9 activity. Unless noted, for the remainder of the studies we used a dose of 6.25 uM Hb (25 uM heme) between 6 and 24 h.

### Hb-induced lipid peroxidation is associated with increased lactate dehydrogenase nucleic acid concentrations

**Fig 3:**
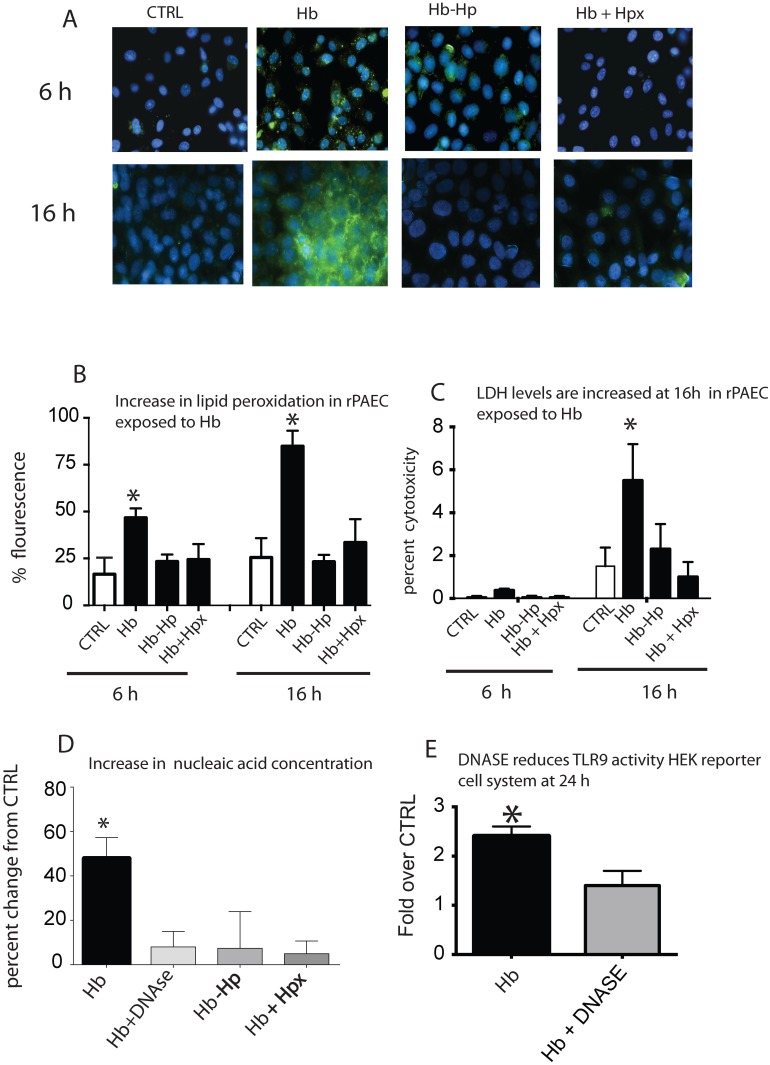
Lipid peroxidation, cell death and nucleic acid accumulation. (A) Photomicrographs that show areas of lipid perioxidation in rPAEC exposed to Hb (25 uM heme) in the presence or absence of haptoglobin or hemopexin. (B) Image J quantification of lipid peroxidation. (C) LDH levels in culture supernatant in rPAEC expose to Hb. (D) Nucleic acid concentrations in culture supernatant exposed to Hb. (E) HEK-blue TLR9 reporter cell activity in cells treated with Hb in the presence or absence of DNASE. *p<0.04 vs. ctrl. Green lipid peroxidation- (White arrows). Hb- hemoglobin Hp-haptoglobin, Hpx- hemopexin.

Cell membrane lipid peroxidation and subsequent cell necrosis may provide a source of uncontained CpG DNA enabling TLR9 activation. Thus, we sought to determine the amount of cellular lipid peroxidation in rPAEC exposed to Hb at either 6 or 16 h, as well as cell culture supernatant LDH levels (as a marker for necrosis) and nucleic acid concentrations. The 6 and 16 h time points were chosen presuming lipid peroxidation and subsequent cell death would precede TLR9 activation. Simultaneously, rPAEC were exposed to Hb-Hp or co-treated with Hb+Hpx to determine if these Hb or heme binding proteins inhibited these Hb-mediated effects. Lipid peroxidation occurred on rPAEC within 6 h and continued to increase at 16 h of Hb incubation (Fig 3A and 3B); we noted increased supernatant LDH and nucleic acid concentrations only at 16 h (Fig 3C and 3D). Lipid peroxidation, supernatant LDH and nucleic acids concentrations were attenuated in rPAEC when incubated with either Hb-Hp or co-treated with Hb+Hpx (Fig 3A–3D).

To further investigate if the rise in nucleic acid concentration in our culture supernatant was linked to TLR9 activity, we treated hTLR9 and HEK-blue TLR9 Null cells with Hb and added a DNase to the culture media. Cells treated with Hb and DNase had lower nucleic acid concentration and TLR9 activity than those incubated with Hb alone (Fig 3D). Co-treatment with either Hp or Hpx also attenuated the nucleic acid concentration and TLR9 activity. Taken together, these data suggest that TLR9 activation in our *in vitro* system most likely occurs in a paracrine fashion; Hb mediated cell necrosis leads to the release of uncontained DNA, thereby activating TLR9 in neighboring live cells.

### Hb-induced TLR9 activation contributes to an increase IL-6 mRNA expression

**Fig 4:**
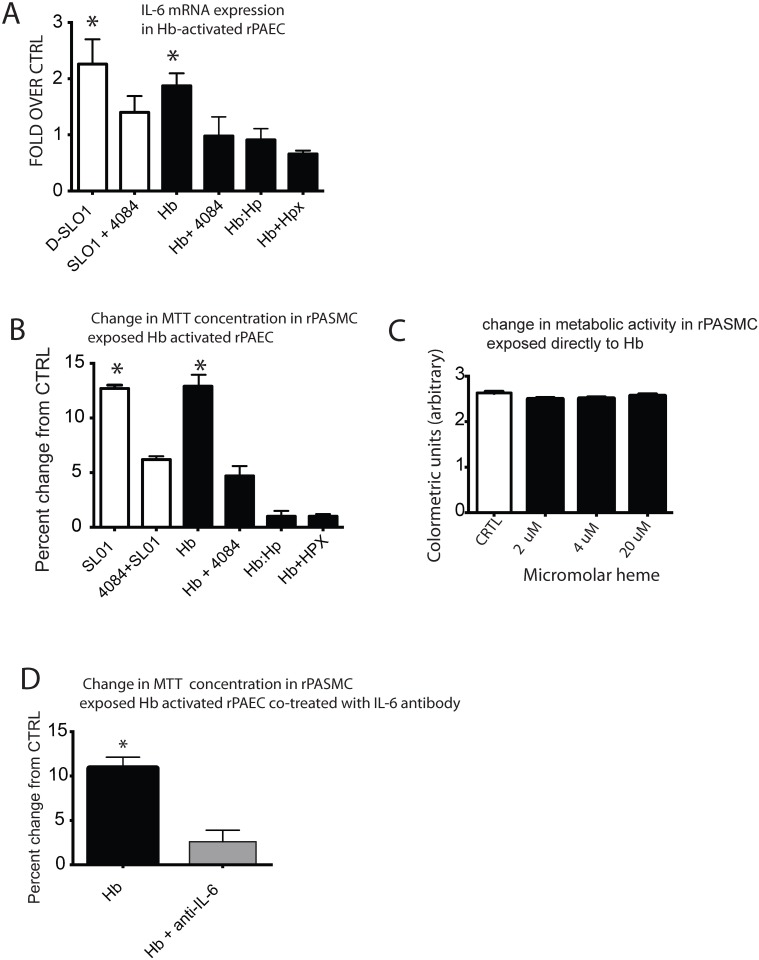
Hb-induced IL-6 via TLR9 and contributes to pulmonary artery smooth muscle cell metabolic activation. **(A)** IL-6 expression in rPAEC exposed to either CpG, or Hb (25 uM heme) in the presence or absence of TLR9 inhibitor 4084, Hp or Hpx. **(B)** Smooth muscle cell proliferation 24 h after exposure to Hb-activated endothelial cells in a co-culture system. Rat PAEC were activated with either CpG or Hb (25 uM) in the presence or absence of TLR9 inhibitor 4084, Hp (Hb-Hp) or Hpx (Hb+Hpx). (**C)** Smooth muscle cell proliferation in rat PASMC that were directly exposed for 24 h to low dose Hb (0.5, 1 and 20 uM heme). **(D)** Smooth muscle cell proliferation at 24 h after exposure to Hb-activated endothelial cells in a co-culture system. Rat PAEC were activated with Hb (25 uM heme) in the presence or absence of an IL-6 antibody. * p ≤ 0.04 vs. ctrl.

To begin to understand how Hb-mediated TLR9 activation may contribute to pulmonary vascular disease, we investigated a TLR9 regulated IL-6 pathway in Hb activated rPAEC. NF-κb is a downstream effector common TLR superfamily [25], and IL-6 is a NF-κb regulated cytokine with pro- inflammatory and pro-growth properties [26, 27]. Our data demonstrate that compared to vehicle control, IL-6 mRNA was increased in rPAEC exposed for 24 h with either CpG DNA or Hb (Fig 4A), and was inhibited when co-treated with the TLR9 antagonist 4084 (Fig 4A). Additionally, the Hb mediated IL-6 response was attenuated in rPAEC exposed to Hb-Hp or cells were co-treated with Hb+Hpx (Fig 4A).

### Hb-induced TLR9 regulated IL-6 pathway and contributes to pulmonary artery smooth muscle cell metabolic activation

It is well established that activated pulmonary artery endothelial cells influence smooth muscle cells [28]. Thus, we utilized a transwell co-culture system that allowed us to culture rPAEC, exposed to either CpG DNA or Hb, with rPASMC in the presence or absence of a TLR9 inhibitor 4084. Additionally, rPAEC were exposed to Hb-Hp or co-treated with Hb+Hpx. Compared to vehicle control, rPASMC had higher metabolic activity when exposed to rPAEC exposed to either CpG DNA or Hb (Fig 4B). This response was attenuated in rPASMC co-cultured with rPAEC either co-treated with 4084, exposed to Hb-Hp, or Hb+Hpx (Fig 4B).

In any transwell co-culture system, solutes added to the culture media in one compartment, such has Hb in the top well, are capable of passive diffusion across the cell monolayer into the adjacent compartment. Thus, we considered whether the increase in metabolic activity of rPASMC observed in our co-culture system was due to a direct Hb effect. Analyses of Hb concentration in the rPASMC compartment revealed only 1 uM Hb (4 uM heme) had diffused through the monolayer at 24 h (Data not shown). Next, the completion of a dose response demonstrated that rPASMC exposed directly to Hb (2–20 uM heme) did not exhibit any higher metabolic activity when compared to cells treated with vehicle control (Fig 4C). Taken together, these data suggest it was the Hb activated rPAEC that influenced rPASMC metabolic activity, not a direct effect of the Hb itself.

Finally to understand if IL-6 contributed to rPASMC activation, in our transwell system, we activated rPAEC with Hb in the presence or absence of an IL-6 neutralizing antibody. Metabolic activity increased only in the rPASMC exposed to rPAEC activated by Hb (Fig 4D), demonstrating endothelial IL-6 influenced metabolic activity in smooth muscle cells.

### Lung IL-6 is attenuated in endothelial specific MyD88 (EC-MyD88^-/-^) and TLR9 (TLR9^-/-^) null mice exposed to chronic Hb infusion

**Fig 5:**
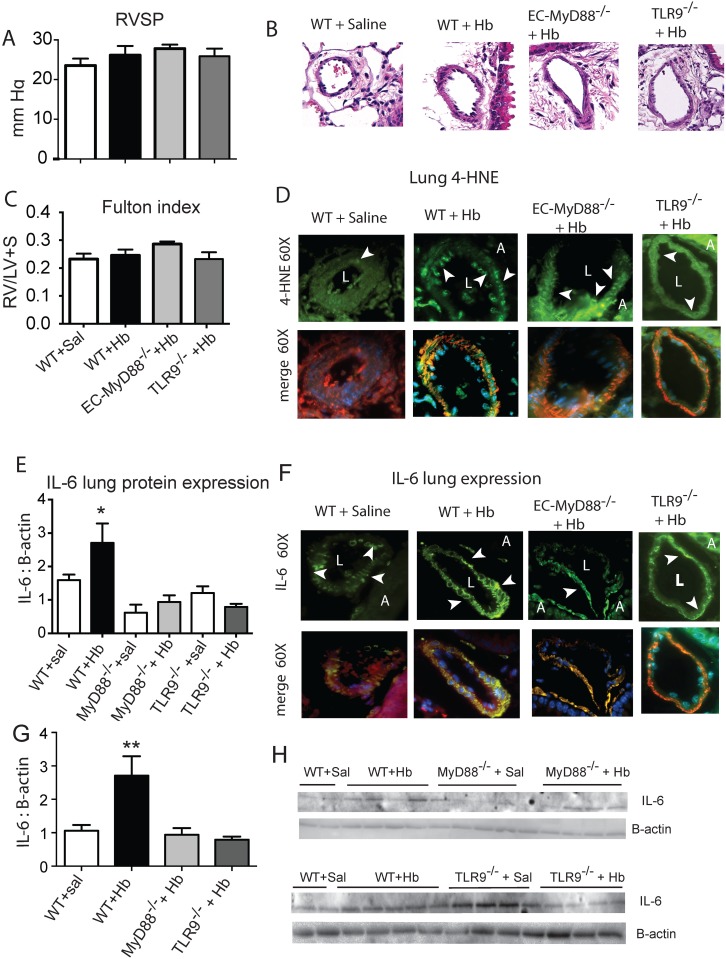
*In vivo* chronic Hb infusion in wild type and endothelial specific MyD88 null mice. (A) Right ventricular systolic pressures after 3 weeks of Hb infusion. (B) Photomicrographs of pulmonary arteries (< 100 u in diameter) stained for haemtoxyline and Eosin (H&E) that show no significant remodeling after 3 weeks of Hb infusion (6 mg/day; n = 6 per group) (C) Fulton index. (D) Photomicrographs of pulmonary arteries (< 100 u in diameter) stained for expression of the lipid peroxidation product 4-HNE and counter stained with alpha smooth muscle actin and DAPA. (E) Western blot of whole Lung IL-6 expression of each group. (F) Photomicrographs of pulmonary arteries (< 100 u in diameter) stained for IL-6 expression and counter stained with alpha smooth muscle actin and DAPA. (G) Western blot of whole lung IL-6 in which all saline infused animals were pooled. (H) Representative western blot of whole lung IL-6. * p = 0.041 vs saline infused WT mice. ** p = 0.002 vs. saline infused mice. White arrows, areas or either 4-HNE or IL-6. Sal- Saline, L- pulmonary artery vessel lumen, A- Airway.

To investigate if our *in vitro* data could be recapitulated *in vivo*, we continuously infused either saline or Hb into the jugular vein of wildtype (WT), EC-MyD88^-/-^, or TLR9^-/-^ mice for 3 weeks. After which, we evaluated mice for evidence of developing pulmonary vascular disease including right ventricular systolic pressures (RVSP), a marker of lung oxidative stress 4-hydroxynonenal (4-HNE), and marker of PH, lung IL-6 concentration. As expected, even after pooling all the saline infused mice, we observed no significant differences in RVSP in either the WT or EC-MyD88^-/-^ or TLR9^-/-^ Hb infused mice (Fig 5A). This data suggests in the absence of other concomitant factors, such as hypoxia, 3 weeks of Hb infusion doesn’t allow sufficient time for mice to develop Hb-mediated pulmonary hypertension. In contrast to RVSP data, we did observe more areas in the lung vasculature that stained positive for 4-HNE in the Hb compared to saline infused mice (Fig 5B). Analysis of whole lung protein content and microscopic evaluation of the lung vasculature revealed the highest IL-6 concentration occurred in the WT Hb-infused mice (Fig 5D–5G). Taken together, these data support our *in-vitro* findings that Hb vascular toxicity can be linked to oxidative stress, endothelial toll-like receptors and/ or global TLR9, and an inflammatory response.

## Discussion

**Fig 6:**
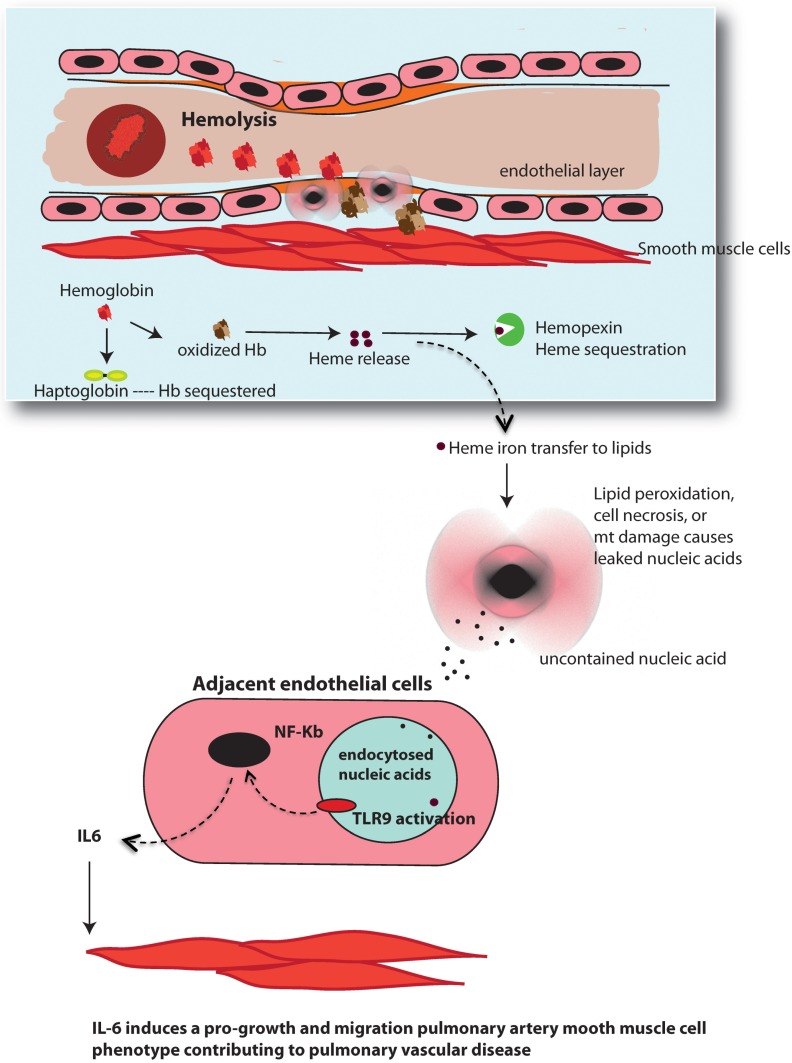
Schematic of Hb-mediated TLR9 pathway. Hb redox reactions cause lipid peroxidation that leads to release of uncontained nucleic acids from either necrotic cell death or excision of damaged mitochondrial (mt) DNA. Uncontained nucleic acid CpG motifs from necrotic cells are capable of activating TLR9 on adjacent endothelial cells after being endocytosed. Alternatively, endothelial cell TLR9 activation may occur autogenously in endothelial cells from damaged mt DNA. TLR9 activation upregulates IL-6 that induces a pro-growth, pro-migration smooth muscle cell phenotype contributing to pulmonary vascular disease.

The principle original finding of the present investigation was that Hb oxidation, cellular lipid peroxidation, and release of uncontained nucleic acids can trigger a TLR9 regulated IL-6 signaling pathway (Fig 6). In addition, these Hb-mediated events were inhibited when either Hb was bound to Hp or cells were co-treated with Hpx, suggesting oxidized Hb and released heme is an initiating event. Additionally, the data afforded from our co-culture model provides evidence that Hb-induced IL-6 upregulation in the endothelial cell contributes to increased metabolic activity in pulmonary smooth muscle cells, indicative of a pro-growth, pro-migration phenotype. Taken together these results suggest a novel pathway by which exposure to free Hb, due to chronic hemolysis, may contribute to pulmonary vascular disease.

In hemolytic disease states, circulating free Hb can persist at concentrations ranging from 10 to 300 uM[29], which depletes the concentrations of the Hb and heme clearance proteins Hp and Hpx, respectively. Once these clearance proteins are overwhelmed, Hb extravasates more easily from the vessel lumen into the vascular wall and elicits numerous biochemical and physiological maladaptations [4, 10]. Of these, the most characterized mechanism is the Hb induced scavenging of NO, leading to persistent vasoconstriction, which plays a key role in the development of pulmonary hypertension associated with sickle cell disease[1, 6–9]. In addition, it is also evident that within this microenvironment, the oxidation of Hb and consequent release of heme, plays a significant role in both organ and vascular pathobiology in hemolytic disease syndromes [21, 30].

Hb and heme toxicity is principally attributed to their pro-oxidative nature, which causes lipid peroxidation leading to endothelial cell damage and cell death [2, 4, 11, 12]. The results presented herein support these previous observations that Hb-mediated increase in lipid peroxidation and elevated LDH in cell supernatant occurred at both a physiologically relevant level of Hb and in a time frame congruent with the oxidation of HbFe^2+^ to HbFe^3+^. As expected, both lipid peroxidation and elevated LDH were attenuated in rPAEC exposed to Hb-Hp or co-treated with Hb+Hpx. It has been previously reported that Hp acts as an antioxidant when it binds Hb by attenuating the redox reactions caused by Hb [31, 32], thereby preventing reactive oxygen species (ROS) from damaging cell membrane lipids [4, 21, 33]. Further, since it is well recognized that lipid peroxidation leads to cell death, it is not surprising that haptoglobin and hemopexin attenuate LDH concentrations. It is notable that Hp and Hpx are equally effective at preventing lipid oxidation and cell cytotoxicity in our model. This suggests that heme is primarily responsible for these initiating events, supporting previously reports that Hb-Hp can prevent heme loss[4, 33] and that Hpx binds heme at substoichiometric concentrations [30].

For TLR9 activation to occur, CpG DNA must first access the intracellular compartment, a process that can occur from either bacterial DNA entering the cell from the outside or bacterial DNA located within the cell [16]. For host DNA, this means either emerging from cells dying, or originating from mitochondrial DNA within the cell that are released due to damage or escape during mitophagy[16, 20, 34]. The increased nucleic acid concentrations we detected in our cell culture supernatant is evidence that Hb-mediated TLR9 in our system is activated by released cellular contents of adjacent cells undergoing necrotic cell death. This is further supported by the observation that TLR9 activation in our TLR9 HEK Blue reporter cells is attenuated with the addition of DNase I to the culture media. As such, we speculate that necrotic cell death could be one of the mechanisms resulting in unregulated digestion of cell components and release of uncontained nuclear or mitochondrial DNA particles acting as DAMPS and interacting with TLR9. However, besides necrosis, several other cell death pathways may be relevant to Hb-mediated TLR9 activity in our system. These include necroptosis, ferroptosis and pyroptosis. Necroptosis and ferroptosis are regulated cell death pathways that can be mediated by heme-triggered oxidative stress [30, 35], and pyroptosis is a Caspase-1 mediated cell death mechanism that is inherently explosive, releasing a host of cytokines and DAMPS such as DNA or ATP into the milieu [36]. In our studies, we did not specifically examine each of these cell death pathways to understand their contribution to the activation of TLR9 by mediating an increase of DNA fragments in our cell culture supernatant acting as DAMPS. However, this will be important to elucidate in future studies.

Evidence in our system suggests Hb-mediated TLR9 activation is likely due to nuclear or mitochondrial DNA (mtDNA) released during cell death and entering adjacent cells from the outside. However, it is plausible that mtDNA originating from inside the cell may also contribute. It has been suggested that severely damaged mtDNA may either be exported from the organelle to the cytoplasm, or may escape the autophagy process that allows for “cell autogenous TLR9 activation” [20]. Relevant to Hb cell toxicity, mtDNA is sensitive to oxidant damage [34, 37]. Thus, it’s reasonable to speculate that Hb, as a strong oxidant, propagates Hb-mediated reactive oxygen species leading, to the excision of damaged mtDNA or autophagy. Further, this may allow cytosolic mtDNA to initiate cell autogenous TLR9 activation. Recently, Chintagri et al. observed damaged or dysfunctional mitochondria in lung alveolar type I cells exposed to higher transition states of ferric Hb (Fe^3+^) and ferryl Hb (Fe^4+^)[5]. We also noted, in our cell culture system, a ~ 15% decrease in active mitochondria in a TMRE flow cytometry assay (data not shown). Taken together, these data suggest the toxic effects of oxidized Hb are not limited to the outer cell membrane, but also effect internal cell organelles including the mitochondria. Relevant to the current study, depending on the severity, mitochondrial damage also leads to cell death. Thus, some cell death in our system may be due to mitochondrial damage potentially contributing to DNA fragments in the supernatant. In the future it will be important to understand how Hb-mediated internal cell damage triggers cell survival or cell death, and how these processes interface with immune receptors including TLR9.

Pulmonary hypertension is a multifactorial disease that affects a disproportionately large percentage of patients who concurrently suffer from hemolytic anemia diseases [7, 38]. PH in this population is generally characterized by pulmonary pressures greater then 25 mm Hg, endothelial dysfunction and proliferation and migration of medial smooth muscle cells [39, 40]. It is recognized that in both rodent models and humans, numerous pathogenic pathways have been associated with IL-6 upregulation. In particular, it has been reported that IL-6 contributes to the vascular remodeling in PH disease [41, 42]. In rPAEC we observed a TLR9 regulated IL-6 pathway, however we are not the first to observe this phenomenon. Previous *in vitro* and *in vivo* studies demonstrate TLR9 can regulate IL-6 in endothelial cells [23, 26]; to our knowledge, we are the first to investigate this pathway in the context of contributing to hemolysis associated pulmonary vascular disease. Our observations utilizing a co-culture system to mimic the interactions between endothelial and smooth muscle cells support the notion that TLR9 activation and up-regulation of IL-6 alters the metabolic activity of smooth muscle cell indicative of a pro-growth, pro-migration phenotype.

*In vivo*, constant Hb infusion increases lipid peroxidation products, inflammatory mediators and contributes to pulmonary vascular disease [21, 43]. In this study Hb increased 4HNE, a product of lipid peroxidation, in the lung vasculature of both Hb-infused WT, EC-MyD88^-/-^, and TLR9^-/-^ mice, but both the EC-MyD88^-/-^ and TLR9- Hb infused mice had less lung IL-6 compared to the WT cohort. This supports our hypothesis that Hb-mediated lipid peroxidation is linked to IL6 through an endothelial TLR mechanism. However, MyD88 is signal transduction protein common to nearly all Toll-like-receptors including TLR9 [44]. Thus, in the current study we can’t rule out the possibility that the attenuated IL-6 response we observed in the lungs of Hb infused EC-MyD88^-/-^ mice was due solely to TLR9. Because Heme either indirectly or directly activates t TLR-4 [45], and Hpx inhibited the effects of oxidized Hb in our *in vitro* systems, it’s possible there is TLR4/TLR9 interaction regulating IL6. In the present study we did not investigate whether TLR4 influenced endothelial IL-6 activation either alone or in conjunction with TLR9.

As expected there was no significant pulmonary vascular remodeling indicative of smooth muscle cell proliferation in any of our strains of Hb-infused mice compared to control cohorts. This is likely due insufficient exposure time of Hb to the vasculature. In previous studies using the Hb-infused rat model, we observed signs of pulmonary vascular disease including a modest rise in mean pulmonary arterial pressures, pulse pressures and medial hypertrophy at 5 weeks or later of Hb exposure in the ambient environment[43]. In those studies, we concluded the interaction between Hb concentration, exposure time, and environmental factors, such as hypoxia exposure, plays a critical factor in the development of Hb mediated pulmonary vascular disease. Our data herein would suggest a similar dose response and time course occurs in the mouse.

### Clinical relevance

Morbidity associated with Hb toxicity is multifactorial and dependent on the disease state or medical condition as well as genetic, and environmental exposure factors unique to each individual. Thus, targeting Hb-mediated vascular effects will require a host of therapeutic options that can be used either alone or in combination. The sequence of events we have described may provide potential therapeutic strategies to combat Hb-mediated pulmonary vascular disease. Our data confirms previous reports that sequestering Hb or heme with Hp or Hpx prevents a multitude of downstream events; however, our data also suggests antioxidant and TLR9 inhibition may serve as alternative approaches. Further, for some individuals suffering from hemolytic disease syndromes, combining these strategies with NO based therapies may provide a cocktail to reverse or stop the progression of pulmonary hypertension. In conclusion, these data implicate a TLR9-IL6 pathway in Hb-mediated pulmonary vascular disease.
