# Correction: Hemoglobin induced cell trauma indirectly influences endothelial TLR9 activity resulting in pulmonary vascular smooth muscle cell activation

**DOI:** 10.1371/journal.pone.0173652

**Published:** 2017-03-06

**Authors:** Zoe Loomis, Paul Eigenberger, Katherine Redinius, Christina Lisk, Vijaya Karoor, Eva Nozik-Grayck, Scott K. Ferguson, Kathryn Hassell, Rachelle Nuss, Kurt R. Stenmark, Paul W. Buehler, David C. Irwin

The tenth author’s name appears incorrectly. The correct name is Kurt R. Stenmark. The eleventh author’s name appears incorrectly. The correct name is Paul W. Buehler.
